# Mr. Knipe, on Amputation

**Published:** 1800-11

**Authors:** William Knipe

**Affiliations:** Garstang


### *To the Editors of the Medical and Physical Journal.*

GENTLEMEN,

I Have thus far with the greatest satisfaction, perused your truly valuable Journal; which seems to me to be of intrinsic utility to the medical world, both from the variety of subjects it contains, and the perspicuity and accuracy which every where attends the execution of it. Should you think the following case of Amputation worthy to be inserted in your Publication, I offer it to your services. And am,

GENTLEMEN,

With great respect, your's truly,

WILLIAM KNIPE.

*Garstang, Oct.* 2, 1800.

---

JOHN SMITH, aged 15, was afflicted with a number of large unequal tumors upon the left hand, which he says originated about ten years ago. His relations defcribe their firft appearance to be fimilar to a number of warts; thefe have gradually increafed in point of fize, which has been very rapid within thefe few laft years.

The appearance of the hand, on account of its fize, was very aftonifhing. The tumors were feveral in number, although fome of them were connected, forming an unequal appearance. The largeft occupied the fubftance of the hand; feveral others, about the fize of oranges, were fituated upon different fingers, which laft were disjointed, the ends of which were placed promifcuoufly upon the top of the different tumors. A number of very large and diftended veins were feen extended over the furface of the hand. The integuments were ulcerated in feveral places, and of a dark livid fhining colour, having much the appearance of fcrophula. The laft two months, his health was much impaired; was affected with hectic fever, along with exceffive perfpiration through the night. His conftitution was emaciated; probably the hectic might be the effect of abforption of aërated matter, together with the debility induced by the quantity of blood requifite for the nourifhment of fo large a tumor. From the fize, fituation, and general appearance of the whole hand, with the impaired habit of the patient, amputation feemed effentially neceffary; removing the tumors by diffection was utterly impracticable.

The operation was performed on July 23, 1800, about two inches above the wrift, by the double incifion. After amputation I examined the hand, the weight of which was five pounds and a half; the circumference of the largeft tumor was exactly twenty-five inches. The appearances upon diffection were as follow: Upon removing the integuments, I found that the large tumor took its origin from the articulation between the carpal and metacarpal bones; this feemed covered on the upper part by a number of tendinous fafciæ; the whole of the tumors were furrounded by a confiderable thicknefs of bone, which was particularly fo at their different origins, where a degree of caries offium was obferved. The bones of the fingers, where the tumors began, were likewife carious, and their perioftium was denuded. The four metacarpal bones were completely obliterated, probably by the abforption of offific matter. The internal part of each tumor confifted of a matter, fimilar to jelly, quite tranfparent, yet not tenacious. The ftump healed in a fortnight, by the firft intention; and the patient had neither ftarting of the ftump, nor fymptomatic fever. I have feen him feveral times fince, and find his health and appearance much improved. It may be worth while to notice, that the left foot has been for feveral years affected with hard fmall

ſmall excreſcences, ſimilar to nodes; theſe, however, remain of a ſtationary ſize. I have ſtated the caſe exactly as it occurred; but being a young practitioner, beg leave to ſuſpend my ideas reſpecting the phyſiological part of ſuch a production, flattering myſelf the ſtatement of the caſe may excite the attention of ſome able phyſiologiſt.

